# Fabrication and Characterization of the Li-Doped ZnO Thin Films Piezoelectric Energy Harvester with Multi-Resonant Frequencies

**DOI:** 10.3390/mi10030212

**Published:** 2019-03-26

**Authors:** Xiaofeng Zhao, Sen Li, Chunpeng Ai, Hongmei Liu, Dianzhong Wen

**Affiliations:** The Key Laboratory of Electronics Engineering, College of Heilongjiang Province, Heilongjiang University, Harbin 150080, China; 2161311@s.hlju.edu.cn (S.L.); 2011026@hlju.edu.cn (C.A.); wendianzhong@hlju.edu.cn (D.W.)

**Keywords:** piezoelectric energy harvester, Li-doped ZnO thin films, MEMS technology, multi-resonant frequencies, load power

## Abstract

A novel piezoelectric energy harvester with multi-resonant frequencies based on Li-doped ZnO (LZO) thin films is proposed in this paper, consisting of an elastic element with three (or more) different length cantilever beam arrays and a piezoelectric structure (Al/Li-doped ZnO/Pt/Ti). The LZO thin films of piezoelectric structure were prepared on Pt/Ti/SiO_2_/Si by using a radio frequency (RF) magnetron sputtering method under certain process conditions. When the LZO thin films were deposited with an LZO target concentration of 5 wt%, the piezoelectric coefficient *d*_33_ was 9.86 pm/V. Based on this, the energy harvester chips were fabricated on a <100> silicon substrate using micro-electromechanical systems (MEMS) technology, and its performance can be measured by fixing it to a printed circuit board (PCB) test substrate. The experimental results show that, when exerting an external vibration acceleration of 2.2 g and a vibration frequency of 999 Hz, the energy harvester can achieve a big load voltage of 1.02 V at a load resistance of 600 kΩ, and a high load power of 2.3 µW at a load resistance of 200 kΩ.

## 1. Introduction

It’s well known that vibration is common in the environment, such as motor operation vibration, bridge vibration, etc. [1,2]. The vibrational energy harvester can convert the energy generated by these vibrations into electrical energy, which can then be used as the power supply of various electronic devices. This is especially applicable in the fields of low energy-consumption electronic devices [3,4]. Nowadays, many different piezoelectric energy harvesters have been fabricated using piezoelectric materials. However, the piezoelectric energy harvesters prepared using the above-mentioned piezoelectric materials have many disadvantages which limit their further development, such as their complicated fabricating process, poor compatibility with micro-electromechanical systems (MEMS) technology, and the need for external power sources. To overcome the above limits, the piezoelectric method with excellent compatibility with MEMS technology has been utilized to prepare different thin films with impurities doping, to simplify the preparation process and improve energy performance. In 2014, Hu, et al. presented a high power Co_3_O_4_/ZnO piezoelectric transducer with a multi-layer (Au/Co_3_O_4_/ZnO/Ti) thin film cantilever beam structure based on the magnetron sputtering method under optimized deposition conditions, achieving an output power 10.9 times higher than that of the ZnO piezoelectric transducer under the conditions of a load resistance of 100 kΩ and a low operating frequency of 37 Hz [5]. In 2014, Janphuang et al. proposed a single piezoelectric energy harvester by using a low-temperature process and a standard microfabrication technology to bond thinned bulk Pb(Zr,Ti)O_3_(PZT) components on a silicon-on-insulator (SOI) wafer, realizing an average power of 1.6 µW under an optimum load resistance of 11.8 kΩ, and an applied acceleration of 0.1 g at a resonant frequency of 100 Hz [6]. Nevertheless, it can be found that all of the resulting vibration energy harvesters operated at a single resonant frequency, unavailable in external environmental vibration frequency conditions. 

In 2006, Shahruz et al. first proposed a design idea of mechanical band-pass filters with large frequency bands for energy scavenging, by appropriately choosing the dimensions of the beams and the mass of proof [7]. To adapt to the widely external vibration frequency applications, researchers have paid much attention to multi-frequency energy harvesters with a large frequency band. For example, in 2013, Jia et al. fabricated a Si-based vacuum packaged MEMS electrostatic harvester (0.278 mm^3^) with a multi-frequency operation, by accessing five orders of parametric resonance, i.e., exhibiting four and five parametric resonance peaks at room pressure and vacuum respectively, when scanning up to 10 g [8]. In 2014, Nelatury et al. proposed a MEMS electromagnetic energy harvester with three dominant peaks at three different resonant frequencies [9]. Meanwhile, in 2015, Wu et al. also reported a multi-resonant wideband energy harvester based on a folded asymmetric M-shaped cantilever, consisting of three proof masses and an asymmetric M-shaped cantilever [10]. Recently, ZnO piezoelectric thin films has been used to prepare piezoelectric energy harvesters, due to its simple preparation process and excellent energy performance compared with other piezoelectric materials, such as PZT and aluminum nitride (AlN) [11,12,13]. Currently, the main methods for preparing ZnO thin films include the sol-gel method, magnetron sputtering method, and chemical bath deposition. Compared to these preparation methods, it is possible to prepare wafer-level ZnO thin films by patterning on a silicon substrate, based on MEMS technology and magnetron sputtering. The existence of defects, such as Zn interstitial atoms or O atom vacancies, during the preparation of pure ZnO thin films results in ZnO thin films representing as n-type semiconductors. Thus, it is possible to change the conductivity of semiconductor materials with the incorporation of certain impurities (Li, Al, Na, Mg, et al.) [14,15,16,17,18]. For example, in 2013, Yuan et al. proposed a ZnO thin-film driven microcantilever for nanoscale actuation and sensing based on the radio frequency (RF) magnetron sputtering method, and got a piezoelectric coefficient *d*_31_ of about 4.66 pC/N, in view of the deflection of the cantilever beam tip [13]. In 2015, Laurenti et al. fabricated a piezo polymeric transducer (PPT) for health monitoring systems in space applications, based on lead-free ZnO thin films prepared using the RF magnetron sputtering method, and achieved a piezoelectric coefficient *d*_33_ of ZnO up to 12.3 pm/V [19]. In 2018, Fortunato et al. prepared piezoelectric thin films of ZnO-nanorods/nanowalls by chemical bath deposition, realizing piezoelectric coefficients *d*_33_ of 7.01 ± 0.33 pm/V and 2.63 ± 0.49 pm/V for the ZnO-nanorods and ZnO-nanowalls, respectively [20]. Based on this, the resistivity and the impedance of ZnO piezoelectric materials can be increased by incorporating a Li impurity, which also improves the piezoelectric properties of ZnO piezoelectric materials. However, due to a lack of suitable technology, the studies on the fabrication technology and characteristics of piezoelectric energy harvesters with multi-resonant frequencies have seldom been published [21]. 

In this study, a Li-doped ZnO (LZO) thin films piezoelectric energy harvester with multi-resonant frequencies was designed, consisting of an elastic element with three (or more) different LZO length cantilever beam arrays and a piezoelectric structure (Al/Li-doped ZnO/Pt/Ti). By building structure models of a piezoelectric energy harvester, the working principle of the proposed energy harvester was investigated. Based on the properties analysis of LZO thin films and micro-electromechanical systems (MEMS) technology, the fabrication process was studied. At a certain external vibration acceleration, the load voltage of a single beam and the load power of an LZO thin films piezoelectric energy harvester at a load resistance were tested. This study lays the foundation for preparing a multi-resonant frequencies energy harvester. 

## 2. Basic Structure and Working Principle

### 2.1. Basic Structure

**Figure 1:**
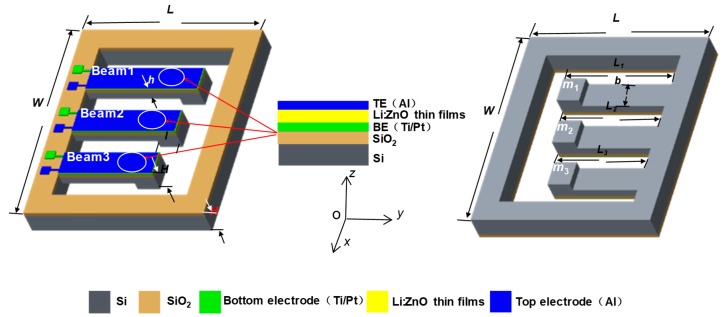
Basic structure of energy harvester with cantilever beam: (**a**) front view; (**b**) back view.

An energy harvester structure based on LZO thin films is given in Figure 1, consisting of an elastic element and a piezoelectric structure Al/Li-doped ZnO/Pt/Ti. As seen in Figure 1a, the elastic element with a cantilever beam array is composed of three (or more) silicon cantilever beams with a mass block on every free end of the three beams, i.e., Beam1, Beam2, and Beam3. The cantilever beam array has different lengths *L*, the same width *W*, and an identical thickness *H*. Figure 1a,b shows the front and back views of the energy harvester, where the lengths of the elastic elements are *L*_1_, *L*_2_, and *L*_3_, respectively (the Beam1 *L*_1_ = 6.25 mm, the Beam2 *L*_2_ = 6 mm, the Beam3 *L*_3_ = 5.75 mm), *b* and *h* are the width and the thickness of each cantilever beam, and *m*_1_, *m*_2_, and *m*_3_ are the mass blocks of the elastic elements. The piezoelectric structure has three layers, including a bottom electrode (BE) layer of the Pt/Ti, LZO piezoelectric thin films and a top electrode (TE) layer of Al.

### 2.2. Working Principle

**Figure 2:**
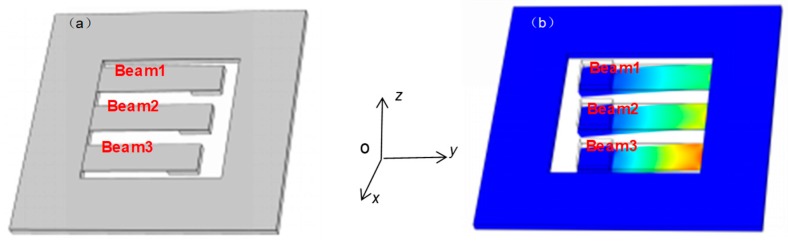
The operating principle of the energy harvester: (**a**) without environmental vibration; (**b**) under environmental vibration.

Generally, the vibration energy harvester has two operating modes—*d*_31_ and *d*_33_. When the vibration energy harvester is under *d*_31_ mode, the top and bottom electrodes of the piezoelectric material (seen inset in Figure 1) are parallel with the plate electrode, accumulating different charges. The piezoelectric material is inflected by the stress along the length of the beam, with a polarization in the direction *z*. As shown in Figure 2a, the energy harvester would not move without environmental vibration, regardless of the gravity acceleration. When there is an environment vibration, as shown in Figure 2b, both the elastic element and the piezoelectric thin films layer of the energy harvester elastically deform based on the piezoelectric effect, resulting in the top and bottom surfaces of the LZO thin films charging in positive and negative directions, corresponding to the conversion from mechanical energy to electrical energy.

When exerting an external environmental vibration on the energy harvester, the vibration would be converted into a force *F* by the mass on the top of cantilever beam array, according to the Newton’s second law. Thus, the *F* can be given as,

(1) $$F=m_{1}a=m_{2}a=m_{3}a,$$

where *a* is the acceleration of an external environmental vibration along the *z*-axis. 

According to the mechanism analysis of the cantilever beams and the small deflection theory [22], the stresses *σ*_1_, *σ*_2_, and *σ*_3_ along the length of the three beams—Beam1, Beam2, and Beam3—can be expressed as,

(2) $$\left\{ \begin{array}{l} \sigma_{1}=\frac{6F}{bh^{2}}\times L_{1}\\ \sigma_{2}=\frac{6F}{bh^{2}}\times L_{2}\\ \sigma_{3}=\frac{6F}{bh^{2}}\times L_{3} \end{array}\right.\;,$$

where *F* is the force generated by the environment vibration. Based on the piezoelectric effect [23,24], the top and bottom surfaces of the LZO thin films accumulate positive and negative charges. The charges *Q*_1_, *Q*_2_, and *Q*_3_ of the cantilever beam arrays are:

(3) $$\left\{ \begin{array}{l} Q_{1}=d_{31}\sigma_{1}\\ Q_{2}=d_{31}\sigma_{2}\\ Q_{3}=d_{31}\sigma_{3} \end{array}\right.\;,$$

where *d*_31_ is the piezoelectric coefficient. By substituting Equation (2) into Equation (3), *Q*_1_, *Q*_2_, and *Q*_3_ of the cantilever beam arrays between the top and bottom electrodes along the *z*-axis are given as:

(4) $$\left\{ \begin{array}{l} Q_{1}=d_{31}\times \frac{6F}{bh^{2}}\times L_{1}\\ Q_{2}=d_{31}\times \frac{6F}{bh^{2}}\times L_{2}\\ Q_{3}=d_{31}\times \frac{6F}{bh^{2}}\times L_{3} \end{array}\right..$$

As shown in Figure 1, the Al/Li-doped ZnO/Pt/Ti piezoelectric structure model is equivalent to a plate capacitor, where piezoelectric structures of the three cantilever beams are equivalent to the three capacitances, *C*_1_, *C*_2_, and *C*_3_, as follows:

(5) $$\left\{ \begin{array}{l} C_{1}=\frac{\varepsilon_{33}A_{1}}{d}\\ C_{2}=\frac{\varepsilon_{33}A_{2}}{d}\\ C_{3}=\frac{\varepsilon_{33}A_{3}}{d} \end{array}\right.\;,$$

where $\varepsilon_{33}$ is the dielectric constant of the LZO thin films, *A*_1_, *A*_2_, and *A*_3_ are the piezoelectric thin film effective areas on the cantilever beam array, and *d* is the thickness of the piezoelectric thin films.

According to the definition of plate capacitor *C*, the *C* is proportional to the voltage and inversely proportional to the charges between the two plate electrodes BE and TE. By combining Equation (4) with Equation (5) to get the charge *Q*, the output voltages *V*_out1_, *V*_out2_, and *V*_out3_ between the BE and TE for the three cantilever beams are given as:

(6) $$\left\{ \begin{array}{l} V_{\mathrm{out}1}=\frac{Q_{1}}{C_{1}}=d_{31}\times \frac{6maL_{1}}{bh^{2}\varepsilon_{33}A_{1}}\\ V_{\mathrm{out}2}=\frac{Q_{2}}{C_{2}}=d_{31}\times \frac{6maL_{2}}{bh^{2}\varepsilon_{33}A_{2}}\\ V_{\mathrm{out}3}=\frac{Q_{3}}{C_{3}}=d_{31}\times \frac{6maL_{3}}{bh^{2}\varepsilon_{33}A_{3}} \end{array}\right..$$

From Equation (5), the vibration energy can be converted into the output voltage using the cantilever beam array with the LZO piezoelectric layer, where the output voltage is proportional to the piezoelectric coefficient at a fixed size of cantilever beam array. In addition, the output voltage is proportional to the length of the cantilever beam, but inversely proportional to the width and the thickness of the beams at a constant piezoelectric coefficient. Based on the above theoretical analysis, it is possible to convert the vibration energy into electrical energy by the proposed structure. Furthermore, the output voltage can be improved by increasing the piezoelectric coefficient of the thin films or adjusting the size of the structure (such as decreasing the width or the thickness of the cantilever beams). 

Based on the natural frequency of the cantilever beam structure, the working frequency *f* of the energy harvester is studied. In ideal conditions, *f* can be expressed as [25]:

(7) $$f=\frac{1}{2\pi }\sqrt{\frac{E}{\rho }\times \frac{h}{L^{2}}}\;,$$

where *E* and *L* are the elastic modulus and the length of the cantilever beam, respectively, *b* and *h* are the width and the thickness of the cantilever beam, and *ρ* is the volumetric mass density of the silicon. 

To make the energy harvester work at multi-resonant frequencies, the energy harvester in this study was designed with a structure composed of three cantilever beams with different lengths of *L*_1_, *L*_2_, and *L*_3_, for which *b* and *h* are the same. From Equation (7), the operating frequency range can be obtained as:

(8) $$f=\frac{1}{2\pi }\sqrt{\frac{E}{\rho }\times \frac{h}{L_{1}{}^{2}}}\sim \frac{1}{2\pi }\sqrt{\frac{E}{\rho }\times \frac{h}{L_{3}{}^{2}}}\;.$$

Therefore, the resonant frequency can be changed by adjusting the length and thickness of the cantilever beams. In addition, by optimizing the size and number of the cantilever beams, it is possible to perform the energy harvester at multiple resonant frequencies and at a large frequency band, simultaneously.

## 3. Fabrication Technology

**Figure 3:**
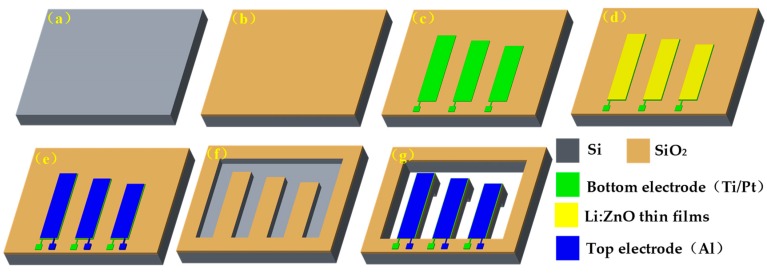
The main fabrication technology process of energy harvester: (**a**) cleaning silicon wafer; (**b**) growing SiO_2_ layer; (**c**) sputtering Pt/Ti bottom electrode layer; (**d**) preparing LZO thin films; (**e**) depositing Al top electrode layer; (**f**) etching chip front side; (**g**) releasing cantilever beam.

Based on MEMS technology, the energy harvester was fabricated on a silicon wafer with <100> orientation. Figure 3 shows the main fabrication process of the energy harvester. As seen from Figure 3, the main steps were as follows: (a) an n-type <100> silicon wafer was cleaned with double-sided polishing; (b) the first oxidation grew a SiO_2_ layer with a thickness of about 300 nm on the silicon substrate, using the thermal growth method; (c) first photolithography, using metal lift-off technology, coated with photoresist, and sputtered a Pt/Ti bottom electrode layer by using magnetron sputtering (JGP-DZS, Shenyang Sky Technology Development Co., Ltd., Shenyang, China), and finally the photoresist was removed; (d) second photolithography prepared LZO thin films by using a radio frequency (RF) magnetron sputtering system (JGP-DZS, Shenyang Sky Technology Development Co., Ltd.); (e) third photolithography and then an Al top electrode layer was developed, using vacuum deposition (ZZ-450A, Beijing Beiyi Innovation Vacuum Technology Co., Ltd., Beijing, China); (f) fourth photolithography, the front side was etched, using inductively coupled plasma (ICP); (g) fifth lithography released the cantilever beam array and formed the cantilever structure with the mass block. 

**Figure 4:**
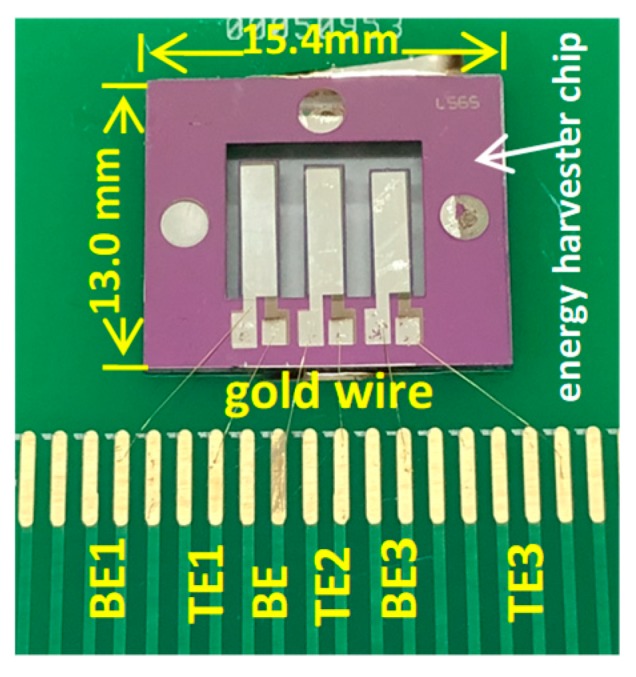
The photograph of the testing structure for the energy harvester.

To study the performance of the proposed energy harvester, the testing structure was designed and fabricated. The main steps were as follows: initially, the testing chip was fixed on a printed circuit board (PCB) with holes, and the cantilever beams were allowed free movement. Thereafter, the proposed chip leads were bonded with an integrated chip press welder (Kullicke & Soffa, KNS4526, Singapore, Singapore). Finally, the solder joints were connected to the chip and the corresponding solder joints on the PCB by six gold wires, including three top electrodes and three bottom electrodes. A photograph of the testing structure for the energy harvester is shown in Figure 4, where the chip (with an area of 15.4 mm × 13 mm) is fixed on the PCB using an integrated chip press welder. 

## 4. Results and Discussion

Since the bottom electrode of the energy harvester chip is Pt/Ti, a Pt/Ti/SiO_2_/Si substrate was used to study the effect of Li doping concentrations on the crystal orientation, piezoelectric coefficient, and surface morphology of LZO thin films. The main preparation processes of the Pt/Ti/SiO_2_/Si substrate were as follows. First, the bottom electrode of Ti was prepared in <100> crystal orientation Si/SiO_2_ using a high-purity Ti target (Beijing Gao Dewei metal, Beijing, China, φ60mm, purity: 99.99%), based on the DC magnetron sputtering method, under the conditions of a deposition time of 5 min, a DC sputtering power of 100 W, and a working pressure of 1.0 Pa. Subsequently, a Ti electrode was grown by using a high-purity Pt target (Beijing Gao Dewei metal, φ60 mm, purity: 99.99%), based on the RF magnetron sputtering under the same conditions described above. Thereafter, the Pt/Ti/SiO_2_/Si substrate was prepared. The LZO thin films were prepared on a Pt/Ti/SiO_2_/Si substrate by adopting RF magnetron sputtering. Under the conditions of a sputtering power of 220 W, a deposition time of 3 h, and a working pressure of 1.0 Pa, the thin films were deposited by using different LZO targets (Beijing Gao Dewei metal, φ60 mm). Finally, three batches of samples for XRD, SEM, and PFM tests were prepared under the same conditions. The sample consists of a bottom electrode layer and a piezoelectric LZO layer.

### 4.1. XRD Analysis

**Figure 5:**
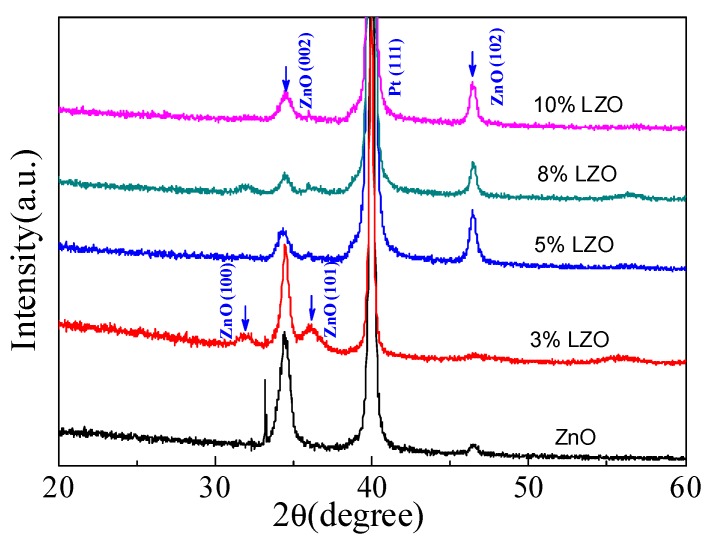
The XRD patterns of the LZO thin films under different Li doping concentrations.

The crystal structure of the LZO piezoelectric thin films with different Li-doping concentrations is characterized by an X-ray diffractometer (XRD, Bruker AXS D8 ADVANCE, Billerica, MA, US). Figure 5 shows the XRD patterns at different Li doping concentrations. As seen in Figure 5, five obvious peaks can be observed, including four ZnO peaks and one Pt peak at 39.9°, corresponding to the (111) characteristic peak of Pt [26]. In Figure 5, the most intense peak belongs to the ZnO (002) plane, indicating the (002) diffraction peak position has been modulated with the increase in Li doping concentrations. At 34.44°, the (002) diffraction peak of the LZO thin films deposited with an LZO target concentration of 5 wt% is close to the characteristic diffraction peak of ZnO [27,28,29]. The weak (100) and (101) diffraction peaks of ZnO mean that the crystallinity of the sample is not high at this time. As the doping concentration increases, the (102) diffraction peak of ZnO becomes stronger compared to the pure ZnO, and is strongest at 5 wt% LZO. With the increase in Li doping concentrations, the (002) diffraction peak intensity initially decreases, and then increases. The above analysis indicates that the ZnO thin films have a perfect c-axis orientation and an excellent crystalline quality. The high c-axis orientation indicates the piezoelectric quality of the ZnO thin films is high [8].

### 4.2. SEM Analysis

**Figure 6:**
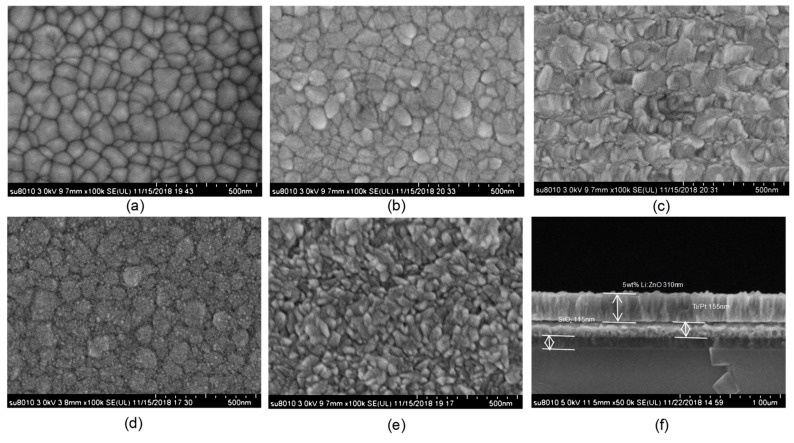
The SEM photographs: (**a**) pure ZnO; (**b**) 3 wt% LZO; (**c**) 5wt% LZO; (**d**) 8 wt% LZO; (**e**) 10 wt% LZO; (**f**) cross-sectional morphology of the 5 wt% LZO.

To observe the surface morphology of the thin films, a scanning electron microscope (SEM, S8010, Hitachi, Tokyo, Japan) was utilized to analyze the particle distribution on the surface of the thin films. In order to enhance the conductivity of the ZnO thin films, the films were treated by gold spray before SEM testing. Figure 6a–e shows the SEM photographs of pure ZnO and LZO thin films with a magnification of 105 times, at different Li doping concentrations. As shown in Figure 6a–c, the surfaces of the pure ZnO and 3 wt% LZO thin films are flat. In contrast, when the doping content was increased up to 5 wt%, large grain gaps were formed on the surface of the LZO thin films. As shown in Figure 6d, the ZnO grain sizes reduced significantly when the doping content was continually increased, confirming the XRD testing results. Figure 6f shows the SEM cross-section morphology of 5 wt% LZO thin films containing three layers, with a marking thickness of LZO thin films of about 310 nm. This indicates that the columnar growth of LZO thin films with a c-axis orientation is perpendicular to the substrate surface, corresponding with the (002) diffraction peak in the XRD analysis.

### 4.3. PFM Analysis

**Figure 7:**
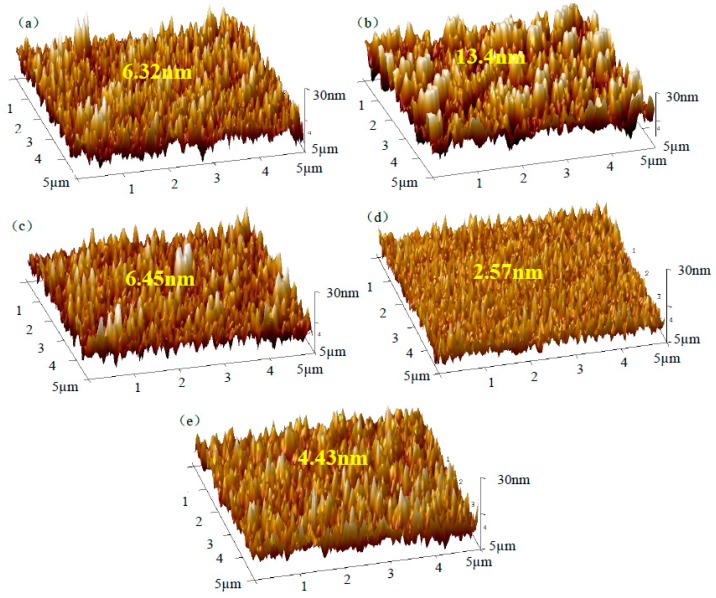
The three-dimensional surface morphology of the LZO piezoelectric thin films under different Li doping concentrations: (**a**) pure ZnO; (**b**) 3 wt% LZO; (**c**) 5 wt% LZO; (**d**) 8 wt% LZO; (**e**) 10 wt% LZO.

To observe the surface roughness and determine the piezoelectric coefficient *d*_33_, the piezoelectric force microscopy test (PFM, Bruker Multimode 8, Billerica, MA, USA) was carried out, where *d*_33_ can be obtained by analyzing the relationship between the displacement of the PFM cantilever tip and the excitation voltage [30]. Figure 7a shows the three-dimensional surface topographies of the pure ZnO piezoelectric films, where the roughness *R_q_* of the pure ZnO thin films is about 6.32 nm. In contrast, when increasing the Li doping concentration up to 3 wt%, the roughness *R_q_* of the ZnO thin films increases up to 13.4 nm, as shown in Figure 7b. When continually increasing the Li doping concentration up to 5 wt%, the *R_q_* is about 6.45 nm—close to that of the pure ZnO thin films in Figure 6c. As seen in Figure 7d, when increasing up to 8 wt%, the *R_q_* is reduced to 2.57 nm. As shown in Figure 7e, when doping up to 10 wt%, the *R_q_* increases to 4.43 nm again.

**Figure 8:**
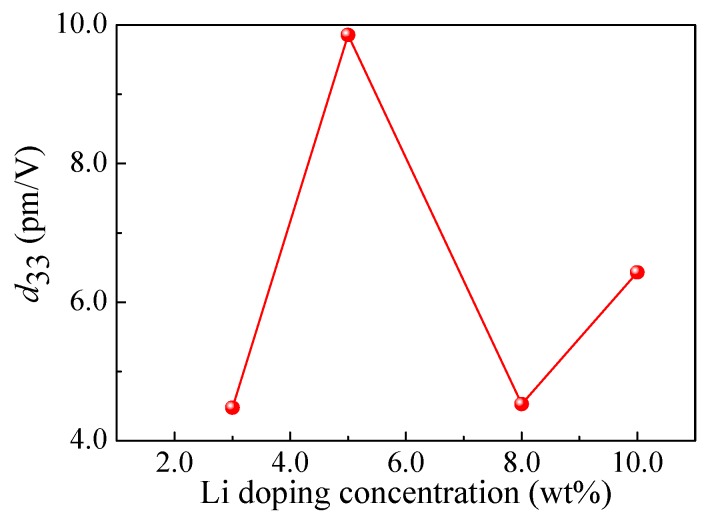
The piezoelectric coefficient *d*_33_ of the LZO thin films under different Li doping concentrations.

The piezoelectric coefficient *d*_33_ was obtained by analyzing the relationship between the displacement of the PFM cantilever tip and the excitation voltage. During the test, the cantilever beam tip was plated on the surface of LZO thin films, where the excitation voltage between the conductive cantilever beam tip and the bottom electrode of the sample was set from 0 V to 10 V. According to the excitation voltage and the probe displacement, the piezoelectric coefficient *d*_33_ can be calculated. Figure 8 shows the relationship curve of the piezoelectric coefficient *d*_33_ and Li doping concentrations. As can be seen from Figure 8, the *d*_33_ goes up to 9.86 pm/V when the Li doping concentration is 5 wt%. Due to the piezoelectric coefficient *d*_33_ being 2–3 times higher than the *d*_31_, the *d*_31_ is very large when the value of *d*_33_ is at its maximum. This indicates that the ZnO thin film with 5 wt% Li doped is the most suitable as the piezoelectric layer of the energy harvester in this study.

### 4.4. Characteristics of the Energy Harvester

**Figure 9:**
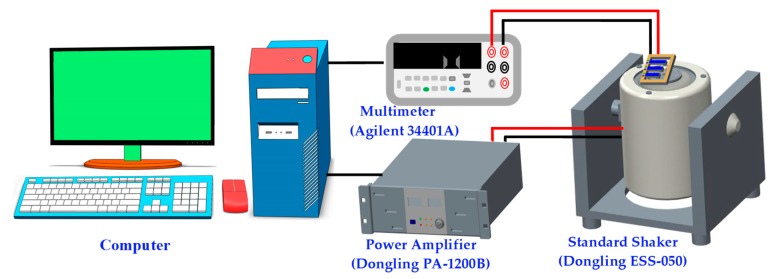
The testing system of the energy harvester.

The automatic data acquisition testing system (exciting frequencies from 50 to 20,000 Hz, and acceleration from 0 to 30 g) used to investigate the vibration characteristics and the response output performance of the energy harvester is shown in Figure 9. This system consisted of a computer, a standard vibration table (Dongling ESS-050, Suzhou, China), a power amplifier (Dongling PA-1200B), and a multimeter (Agilent 34401A, Santa Clara, CA, USA). 

**Figure 10:**
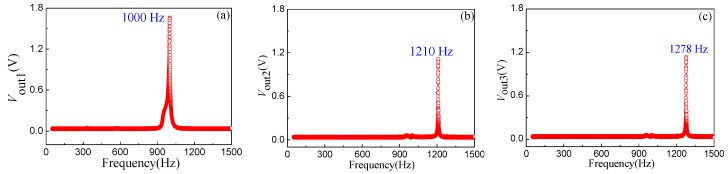
The vibration characteristics of the cantilever beam array: (**a**) the first beam; (**b**) the second beam; (**c**) the third beam.

At room temperature, the chip of the energy harvester was attached on the surface of the standard shaker with a rigid connection, with an external vibration acceleration *a* acting on the energy harvester along the *z*-axis. When applying an external vibration acceleration of 1.5 g and setting an excitation frequency from 50 to 1500 Hz, it is possible to investigate how the frequency influences the output voltages, including *V*_out1_, *V*_out2_, and *V*_out3_, between the top and the bottom electrodes of the three cantilever beams. Figure 10a–c shows the relationship curves between the output voltage and excitation frequency of Beam1, Beam2 and Beam3, respectively. The experimental results show that the maximum output voltages of the three cantilever beams achieves the corresponding resonant frequencies of 1000 Hz, 1210 Hz, and 1278 Hz, respectively. According to Equation (8), the resonant frequency of the cantilever beam decreases as the beam length increases. The experimental results are consistent with the above theoretical analysis.

**Figure 11:**
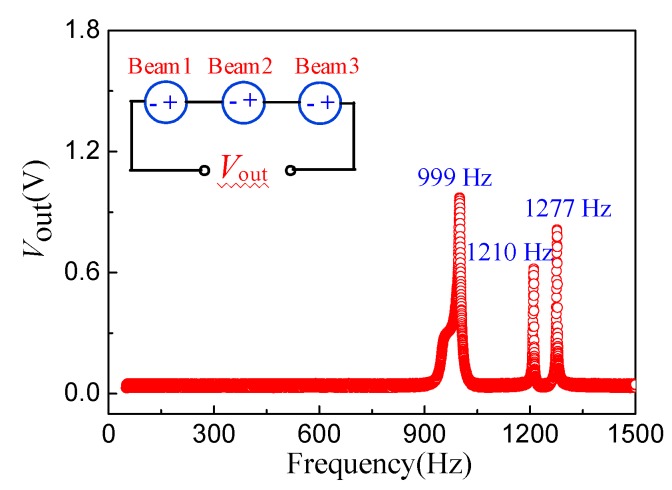
The vibration characteristics of energy harvester series connection.

To get the frequency characteristics of the energy harvester, the bottom and top electrodes of the three cantilever beams are connected in series with each other. Figure 11 shows the vibration characteristics of the proposed energy harvester series connection when applying an external excitation frequency of 50 to 1500 Hz and an acceleration of 1.5 g. The test results show that, due to the existence of three apparent resonant peaks corresponding to the resonant frequencies of Beam1, Beam2, and Beam3, it is possible for the energy harvester to measure the multi-resonant frequencies. The inset illustration in Figure 11 is the equivalent circuit of the energy harvester; each energy harvester with a single cantilever beam can be equivalent to a voltage source. However, the interaction of the three cantilever beams with the series connection causes an increase in the total resistance of the chip, resulting in a smaller output voltage after a series connection than that of a single beam. In addition, by optimizing the size and increasing the number of cantilever beams, the working frequency band of the energy harvester can be improved as well.

**Figure 12:**
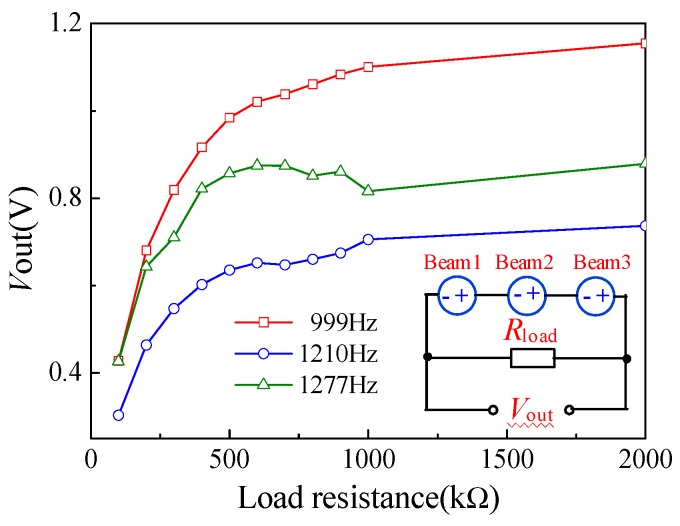
The relationship curves between the load voltage and load resistance of the piezoelectric energy harvester with series connection (inset is equivalent circuit).

**Figure 13:**
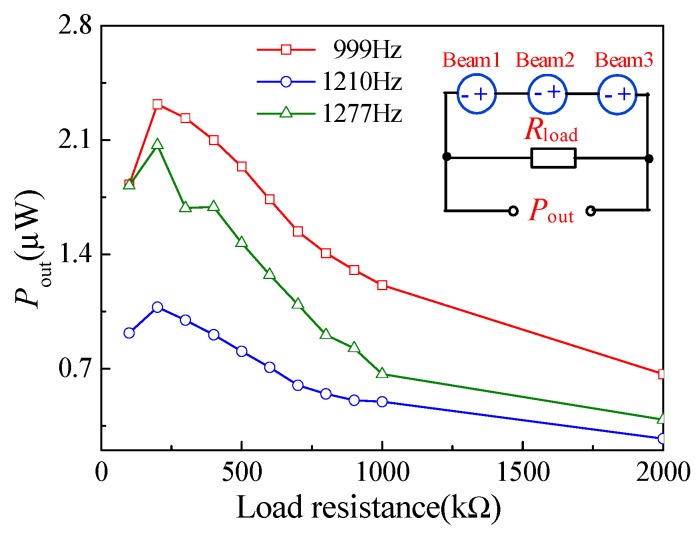
The relationship curves between the load power and load resistance of the piezoelectric energy harvester with series connection (inset is equivalent circuit).

Based on this, the effect load resistance corresponding to the load voltage and power was tested at an excitation frequency the same as the resonant frequency of each cantilever beam. The inset illustration in Figure 12 shows the test circuit connecting a load resistor in series with the energy harvester to measure the load voltage and power across the resistor. Figure 12 shows the relationship curves between the load voltage and load resistance of the piezoelectric energy harvester at three resonant frequencies and an input acceleration of 2.2 g, where the output voltages are different from those in Figure 10 due to the loosening of the energy harvester after multiple measurements, caused by the non-rigid connection between the energy harvester and the standard vibration table. In addition, the output voltage increases first and then remains almost constant with the load resistance. This indicates that the maximum load voltage of the energy harvester is 1.02 V under a load resistance of 600 kΩ, when it is excited by an external vibration with a frequency of 999 Hz and an acceleration of 2.2 g. The load power can be calculated according to the load voltage and the load resistance. Figure 13 shows the relation curves between the load power and load resistance of the piezoelectric energy harvester under the same resonance and acceleration described above, while the inset shows the equivalent circuit of the energy harvester with the load resistance. As shown in Figure 13, the maximum load power for the energy harvester is 2.3 µW at a load resistance of 200 kΩ, when excited by the same vibration. The different output voltages and output power consumptions attribute not only to the different collector structures but also the interaction caused by the series connection between the three collectors. On the basis of the comparative analysis of Figure 11, Figure 12 and Figure 13, the energy harvester achieves its maximum output voltage, load output voltage, and load output power at 999 Hz. In contrast, the output voltage, load output voltage, and load output power are very small at 1210 Hz. In recent years, the energy harvester was designed and fabricated by different piezoelectric materials, such as AZO, ZnO, and PZT. Table 1 compares the piezoelectric energy harvesters. In this study, using MEMS technology, a novel piezoelectric energy harvester with multi-resonant frequencies based on LZO thin films was designed and fabricated. The results show that the proposed energy harvester can achieve multiple resonant frequencies energy harvesting.

## 5. Conclusions

In summary, a novel LZO thin films piezoelectric energy harvester with multiple resonant frequencies was designed in this study. The effects of the Li doping concentration on the crystal structure, piezoelectric coefficient, and surface morphology of the piezoelectric thin films were analyzed. When the LZO thin films were deposited with a concentration of 5 wt% LZO target, using RF magnetron sputtering under certain process conditions, a good crystalline quality and a higher piezoelectric coefficient of the thin films could be achieved, improving the performance of the energy harvester. Based on MEMS technology, the energy harvester with multiple resonant frequencies was fabricated on a silicon wafer. The test results show that it is possible for the energy harvester to realize three resonant frequencies of the cantilever beam array at 999 Hz, 1210 Hz, and 1277 Hz. When the external vibration acceleration is 2.2 g and the vibration frequency is 999 Hz, the largest load voltage is 1.02 V at a load resistance of 600 kΩ, and the maximum load power is 2.3 µW at a load resistance of 200 kΩ. This study of the cantilever beam array lays the foundation for wafer level fabrication of energy harvesters and provides a new strategy to realize the measurement multiple resonant frequencies of piezoelectric energy harvesters in the future.

## Figures and Tables

**Table 1:** The research status of the piezoelectric energy harvester.

Structure	Piezoelectric Material	Performance			Reference
		Resonant Frequency	Maximum Output Voltage	Maximum Output Power	
Cantilever beam	AZO thin films	7.77 MHz	1.61 V	-	[17]
Cantilever beam	Co_3_O_4_/ZnO thin films	37 Hz	-	10.4 μW	[5]
Cantilever beam	ZnO thin films	1300.1 Hz	2.06 V	1.25 μW	[11]
Beam	ZnO thin films	403.8 Hz	10 mV	-	[12]
		489.9 Hz	15 mV		
Cantilever beam	PZT thin films	608 Hz	898 mV	2.16 mW	[31]
Cantilever beam	PZT thin films	36 Hz	-	0.53 μW	[32]
Cantilever beam	PZT thin films	16 Hz	-	740 μW	[33]
Multi-cantilever beam	LZO thin films	999 Hz	1.02 V	2.3 µW	In this work
		1210 Hz	-	-	
		1277 Hz	-	-	
